# Genetic Predisposition to Autoimmune Diabetes Mellitus

**DOI:** 10.32607/actanaturae.27704

**Published:** 2026

**Authors:** Y. V. Dvoryanchikov, I. R. Minniakhmetov, D. N. Laptev, R. I. Khusainova, M. V. Shestakova, N. G. Mokrysheva

**Affiliations:** Endocrinology Research Centre, Moscow, 117036 Russia

**Keywords:** type 1 diabetes mellitus, genetic predisposition, epigenetic modifications, trigger mechanisms

## Abstract

Type 1 diabetes mellitus (T1DM) is a multifactorial disease wherein a genetic predisposition, upon exposure to environmental factors, triggers seroconversion and subsequent beta-cell destruction. The global incidence of T1DM has risen in recent decades, underscoring the significant role environmental factors play in actuating inherent genetic risk. However, the absence of an identifiable unique triggering factor complicates the identification of risk groups. Advances in bioinformatics and epigenetic research are opening new opportunities for early diagnosis, prevention, and novel therapies for the condition. A comprehensive analysis of complex molecular pathways will make it possible to develop algorithms for the early diagnosis, disease course prediction, and therapy personalization based on a patient’s genetic profile. This review systematizes current data on the genetic and epigenetic heterogeneity of autoimmune diabetes and its potential triggers. It assesses the regional and ethnic disparities in T1DM incidence globally and within the Russian Federation, and it discusses the potential of clinical-genetic models for disease prediction

## INTRODUCTION

Type 1 diabetes mellitus (T1DM) is a severe disease with significant societal impact whose prevalence continues to grow globally. According to the Federal Diabetes Register of the Russian Federation, by 2024, the prevalence had reached 194.2 cases per 100,000 adults, compared to 146 per 100,000 in 2010 [1]. Data from the clinical and epidemiological monitoring database of diabetes mellitus as of January 01, 2024, indicate that there are 290,700 people with T1DM in Russia, accounting for 5.7% of all diabetes mellitus (DM) cases [2].

A hallmark of T1DM is a strong genetic predisposition, the clinical manifestation of which depends on exposure to environmental triggers. This interaction initiates an autoimmune process, characterized by seroconversion (the appearance of autoantibodies) and followed by the destruction of pancreatic β-cells. The strongest genetic associations have been centered around the HLA-locus (the major histocompatibility complex) and polymorphic variants in the promoter region of the insulin (INS) gene. Nevertheless, only 5% of individuals carrying high-risk HLA haplotypes and 10% of carriers of risk-associated INS alleles go on to develop overt T1DM within the first two decades of life [3].

The highest genetic risk for T1DM is tied to specific alleles of the HLA-DR and HLA-DQ genes within the HLA locus [4]. The primary risk haplotype, DRB1*03:01–DQA1*05:01–DQB1*02:01, often designated as DR3-DQ2.5, or simply DR3, demonstrates a positive association with T1DM in nearly all studied populations, although the effect’s magnitude varies. The second most significant haplotype DR4 encompassing various DRB1*04:XX alleles exhibits considerable allelic diversity, which complicates precise risk stratification (Table 1).

**Table 1:** Comparison of the risk and protective HLA haplotypes in Caucasian and Mongoloid populations [5]

Haplotype	DRB1-DQA1-DQB1	Race	OR	p-value
DR3	03:01-05:01-02:01	Caucasian	3.64	2 × 10−22
		Mongoloid	10.08	0.0009
DR4	04:02-03:01-03:02	Caucasian	3.63	3 × 10−4
		Mongoloid	3.83	0.5216
	04:03-03:01-03:02	Caucasian	0.27	0.017
		Mongoloid	0.46	0.3772

Genome-wide association studies (GWAS) have helped identify over 100 of the genetic loci associated with the risk of developing T1DM [6]. Notably, approximately 90% of these associated variants reside in noncoding regions of the genome, thus underscoring the critical role that gene expression regulation plays in the disease etiology. The lack of complete concordance for autoimmune diseases, even among monozygotic twins, further highlights the contribution of epigenetic factors.

While most T1DM cases present a characteristic clinical picture that does not require a complex differential diagnosis, a subset of patients exhibit atypical manifestations, which can complicate the diagnosis. Modern molecular genetic methods are employed with increasing frequency to refine diabetes classification and assess genetic risk. For instance, next-generation sequencing (NGS) enables high-resolution HLA allele typing, facilitating the identification of novel risk and protective alleles [7]. Exome sequencing enables detection of rare variants in the genes responsible for monogenic forms of diabetes, paving the way for personalized treatment and monitoring strategies [8]. GWAS and whole-genome sequencing are powerful tools for investigating the polygenic architecture of T1DM, revealing novel risk variants and implicating the biological pathways not previously considered relevant to its pathogenesis. The functional characterization of noncoding variants, combined with insights from single-cell technologies, is rapidly expanding our understanding of the molecular mechanisms underlying T1DM, which is crucial in guiding future research [6]. Nevertheless, the predictive value of known genetic markers for T1DM remains a subject of debate, which underscores the need for further large-scale studies that would take into account the populationspecific genetic structure of diverse ethnic groups worldwide.

This review focuses on the regional and ethnic variations in T1DM prevalence and incidence, synthesizes current knowledge on genetic and epigenetic markers and triggering factors, and tackles the complex polygenic nature of the disease through the lens of molecular genetic analysis and population genetics.

## CLINICAL FEATURES OF TYPE 1 DIABETES

**Fig. 1:**
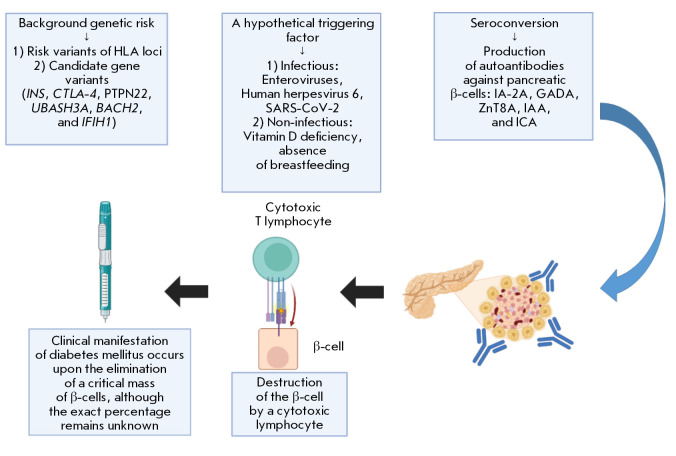
An abbreviated concept of the development of T1DM according to the theory of G.S. Eisenbarth [10]. The authors created the original figure using BioRender and Microsoft PowerPoint (2016)

T1DM is a polygenic, multifactorial disorder characterized by immune-mediated or idiopathic destruction of pancreatic β-cells, leading to absolute insulin deficiency. A background genetic risk, in combination with exposure to environmental factors, triggers autoimmune processes. These processes are clinically manifested by the presence of two or more autoantibodies: against islet cells (ICA), insulin (IAA), tyrosine phosphatase (IA-2A), glutamic acid decarboxylase (GADA), and zinc transporter 8 (ZnT8A) [9] (Fig. 1).

The autoimmune pathogenesis of T1DM is substantiated by the presence of specific autoantibodies [11], along with autoreactive CD8^+^ T cells, CD20+ B cells, CD4^+^ T cells, and CD68^+^ macrophages infiltrating pancreatic islets [12]. It was previously hypothesized that a quantitative defect in the regulatory T-cell (Treg) pool, which is responsible for blocking the effector T-cell response and thwarting autoimmunity through the regulation of the T-cell population size and differentiation, was the key mechanism. However, recent evidence points primarily to qualitative, rather than quantitative, changes in the Treg function [13, 14].

When T1DM is diagnosed in two or more first-degree relatives, these cases are classified as nuclear families (familial T1DM). The prevalence of such familial forms is highest in several countries, including Qatar (14.6%), Oman (22%), and Kuwait (33%). Although these studies did not include genetic association analyses, a recent investigation in Qatar identified the following predominant HLA alleles in patients from nuclear families: HLA-F*01:01:01G, HLA-DPA1*01:03:01G, HLA-DRB3*02:02:01G, HLA-E*01:01:01G, and DRB4*03:01N [15]. This specific set of alleles is not associated with typical T1DM risk, a potential indication of distinct genetic associations within familial T1DM forms. Furthermore, the search for variants in the genes associated with maturityonset diabetes of the young (MODY) revealed that 10% of patients were carriers of variants of uncertain significance or benign variants. Given the presence of autoimmunity and the absence of pathogenic mutations, a MODY diagnosis could be ruled out in these cases [15].

**Table 2:** HLA-alleles and candidate genes associated with predisposition to LADA and the frequency of their occurrence in patients

Gene/Locus	Allele/SNP	Frequency in LADA, %	OR	Reference
HLA-DQA1	rs9272346	68.6	1.45 (1.43–1.48)	[17]
HLA-DQB1	rs9273368	50	3.115 (2.85–3.4)	[18]
INS	rs689	80	1.48 (1.36-1.61)	
SH2B3	rs7310615	55	1.28 (1.19-1.38)	
PTPN22	rs2476601	16.9	1.52 (1.29–1.79)	[19]
CTLA-4	rs231775	49.2	1.39 (1.11–1.74)	

Latent autoimmune diabetes in adults (LADA), which occupies an intermediate position between classic T1DM and type 2 diabetes (T2DM), is a condition deserving of particular attention. According to the current classification outlined by the Immunology of Diabetes Society (IDS), LADA is defined by the following diagnostic criteria: patient age over 30 years, presence of autoantibodies against pancreatic β-cells, and no requirement for insulin therapy for at least six months after the initial diagnosis. LADA accounts for approximately 2–12% of all diabetes cases. Multicenter studies have demonstrated that between 4 and 14% of patients initially diagnosed with T2DM are subsequently found to have LADA upon antibody testing [16]. Therefore, LADA is a clinically and immunologically heterogeneous condition necessitating a distinct approach to diagnosis and management (Table 2).

The situation with LADY diabetes (latent autoimmune diabetes in the young) is less understood: there are no universally accepted diagnostic criteria or comprehensive statistics. According to the available data, patients with LADA are characterized by the presence of autoantibodies to pancreatic β-cells. The disease manifests at a young age (under 30), and the decline in islet cell function and C-peptide levels occurs more rapidly than in type 2 diabetes, which necessitates earlier insulin therapy initiation. The literature discusses an increased frequency of anti-thyroid autoantibodies in males with LADY, and the presence of a high titer of GADA autoantibodies significantly increases the risk, placing these patients in a high-risk group for autoimmune thyroiditis [20]. According to published data, the prevalence of LADY varies significantly across different ethnic groups and age cohorts: from 10 to 75% among European-origin patients with a juvenile phenotype and about 12% among Asianorigin patients [21]. This variability is explained by the lack of unified diagnostic criteria and differences in research methodology. There are limited data on the HLA-region structure in patients with LADY, and, to date, the presence of two haplotypes is known: DRB1*03:01~DQA1*05~DQB1*02:01 (HLA-DR3) and DRB1*09:01~DQA1*03~DQB1*03:03 (HLA-DR9) [22].

It is important to consider that clinical presentation of diabetes in young patients may be caused not only by autoimmune but also by monogenic forms of the disease. The best-known group of monogenic diabetes is MODY (maturity-onset diabetes of the young), caused by mutations in the various nuclear genes responsible for β-cell function. MODY is inherited in an autosomal dominant manner, has different clinical manifestations, and often does not require insulin therapy in the early stages. In addition to MODY, rarer genetic variants, such as mitochondrial diabetes with deafness (MIDD), caused by the m.3243A>G mutation in the MT-TL1 gene of mitochondrial DNA, are also classified as monogenic forms. MIDD is characterized by impaired insulin secretion and sensorineural deafness.

Thus, type 1 diabetes is a clinically and genetically heterogeneous group of diseases. The improvement in molecular genetic diagnostics facilitates the identification of a wide spectrum of rare forms, and this may lead to revision of the current diabetes classification in the future.

## PREVALENCE AND INCIDENCE OF TYPE 1 DIABETES

The analysis of epidemiological patterns, including regional and ethnic variations, as well as patient sex and age, helps identify the associations and potential risk factors of T1DM development, although these data do not always indicate causality. Previously termed “juvenile diabetes,” T1DM was once considered one of the most common chronic childhood conditions; however, disease onset can occur at any age.

Globally, T1DM prevalence remains an epidemiological riddle, with significant disparities even between neighboring regions. For instance, the incidence rate in Estonia is less than one-third of that in Finland, despite their geographical proximity (less than 120 km apart) and shared Finno-Ugric ethnic background [23].

A large multicenter prospective study spanning 25 years (1989–2013) across 22 European countries documented a substantial increase in T1DM incidence among children and adolescents in nearly all regions. The only exceptions were two centers: Catalonia (Spain) and Marche (Italy). The most rapid increase was observed in Katowice (Poland), reaching 6.6% per year. Across Europe, the average annual increase in incidence was 3.4% (95% confidence interval (CI): 2.8–3.9%).

**Table 3:** Countries with > 200,000 T1DM cases in 2024 (excluding Russia)^*^

Country	Total number of T1DM patients	Country population, per thousand population	Regional and ethnic characteristics with references
USA	1,476,859	345,427	An increase in incidence has been observed in the Mountain West (Arizona, Colorado, Idaho, New Mexico, Montana, Utah, Nevada, and Wyoming) and East South Central (Alabama, Kentucky, Mississippi, and Tennessee) regions [29]. Regarding ethnic disparities, the highest frequency is noted among the Caucasian population, followed by African Americans, Hispanics, and other ethnic minorities. Recently, an increase in new T1DM cases among African Americans has been reported [30].
India	940,840	1,450,936	Data on T1DM incidence in India are limited; however, a relatively high incidence is observed in South India (Karnataka and Tamil Nadu) compared to the North (Haryana) [31]. Studies on ethnic differences are scarce and require further research.
China	598,906	1,419,321	The highest incidence is noted in the Northeast (Heilongjiang, Liaoning provinces) and North. The primary inhabitants of these provinces are Han Chinese [32, 33]. Unfortunately, data from the studies on epidemiological and ethnic differences are limited due to uneven access to healthcare in China.
Brazil	499,902	211,999	The highest prevalence is observed in the southern states (Paraná, Rio Grande do Sul) and the southeastern state of São Paulo [34]. T1D is most frequently diagnosed in individuals of Caucasian descent, followed by Pardo (Brazilians of mixed ancestry), Afro-Brazilians, and indigenous peoples [35, 36].
Great Britain	340,794	69,138	The highest incidence is noted in the North (Scotland), the East (Yorkshire and the Humber), and the West Midlands region (Birmingham) [37]. The vast majority of T1D cases affect individuals of Caucasian ethnicity, followed by those of Mongoloid ethnicity (excluding Chinese), and individuals of Negroid ethnicity [38].
Germany	336,936	84,552	The highest incidence is observed in the west (Ludwigslust-Parchim district) and east (Vorpommern-Greifswald district) of Mecklenburg-Vorpommern, as well as in the west (Leer, Oldenburg districts), southwest (Emsland district), and northwest (Aurich, Ammerland districts) of Lower Saxony [39]. Most T1D patients are ethnic Germans, followed by individuals of Eastern European, Southern European, and Turkish descent [40, 41].
Canada	243,390	39,742	The highest incidence is noted in the northwest of Canada (Newfoundland and Labrador province) [42]. The majority of T1D patients are of Caucasian descent, while minorities include Indigenous peoples of Canada and individuals from Pacific Island nations [43].
Saudi Arabia	222,942	33,963	Data on T1D incidence are limited; the few available studies indicate a higher frequency in the eastern part of the country (Dhahran) [44]. Regarding ethnic differences, Arabs and Bedouins are more susceptible to T1D [45].

^*^Data sources: The IDF Diabetes Atlas (prevalence - https://diabetesatlas.org/data-by-indicator/type-1-diabetes-estimates/people-with-type-1diabetes-all-age-groups) and UN Data (population - https://data.un.org/default.aspx).

The rate of increase was similar between boys and girls in the 0–4 and 5–9 age groups. However, in the 10–14 age group, the incidence rose more rapidly in boys. A four-year cyclicity was noted in several centers, with the last peak occurring in 2012, although the mechanism underlying this phenomenon remains unexplained [24]. Subsequent years have continued to show this overall upward trend in incidence among children and adolescents, as confirmed by more recent epidemiological reviews [25] (Table 3).

In the United States, according to the 2021 National Diabetes Statistics Report, 1.7 million American adults (5.7% of all adults with diagnosed diabetes) have T1DM and require insulin therapy [26]. The SEARCH study, conducted across five U.S. medical centers (2002–2017), reported an increasing incidence of T1DM among the youth across ethnic groups: 4.84% in Asian/Pacific Islander, 4.14% in Hispanic, and 2.93% in non-Hispanic Black youth. Incidence among children went from 19.5 per 100,000 in 2003 to 22.2 per 100,000 in 2018. The peak incidence occurs at age 10 for children aged 0–19 years and at age 16 for adolescents aged 10–19 years. The peak occurs later in boys (age 12) than in girls (age 10), and earlier in Hispanic and Asian youth compared to other ethnic groups [27]. Recent data confirm that these trends persist, notwithstanding regional fluctuations [28] (Table 3).

Within the Russian Federation, significant regional variability in prevalence is observed. In 2024, the highest rates were recorded in regions of the European North (Arkhangelsk and Vologda region, Republic of Karelia), exceeding 275 cases per 100,000 adults [2]. One hypothesis for this high prevalence is historical ethnic admixture with Finno-Ugric populations, among whom T1DM frequency is also elevated.

The lowest prevalence was observed in the Eastern Caucasus and Caspian regions, particularly in the Chechen Republic (61.6 cases per 100,000 adults). Low rates were also recorded in the Republic of Sakha (Yakutia), Khabarovsk region, and the Jewish autonomous region (100–124 cases per 100,000 in 2024). The lowest registered prevalence was in the Chukotka autonomous region (43.9 cases per 100,000); however, these data have not been updated since 2019, complicating comparisons with other regions [2].

In conclusion, both the global and regional epidemiology of T1DM are characterized by a significant heterogeneity, underscoring the necessity for a comprehensive investigation of the factors influencing disease distribution, including the genetic, ethnic, and environmental determinants.

## THE ROLE OF ENVIRONMENTAL FACTORS IN REALIZING THE GENETIC RISK OF TYPE 1 DIABETES

Environmental factors act as triggers that promote the realization of genetic predisposition to T1DM, as evidenced by the substantial increase in incidence over recent decades [25]. The dynamics of this increase cannot be explained by changes in the frequency of inherited genetic variants, which has remained relatively stable. The fact that concordance for autoimmune diseases in monozygotic twins is less than 100% (ranging from 13 to 61%), coupled with accumulated data on the epigenetic mechanisms of gene expression regulation, underscores the importance of further investigating the influence of external factors on the actuation of inherent genetic risk.

Several epidemiological studies [46, 47] have identified past viral infections as primary potential triggers. These include human enterovirus A (e.g., Coxsackievirus A4, A2, A16, Enterovirus A71), human enterovirus B (e.g., Echovirus, Coxsackievirus B), rubella virus, cytomegalovirus, mumps virus, rotavirus, human herpesvirus 6 (HHV-6), parvovirus B19, influenza virus (e.g., H1N1), and the SARS-CoV-2 infection [48, 49].

The first proposed mechanisms by which viruses may trigger an autoimmune response are molecular mimicry, epitope spreading, bystander activation, and the bystander effect [50]. The results from retrospective and cohort studies assessing the role of upper and lower respiratory tract infections of various etiologies remain contradictory. That is likely to be due to the insufficient molecular verification of pathogens, which limits the ability to identify the specific infectious agents associated with T1DM development [46].

However, enteroviruses have garnered particular attention, as children with T1DM are found to more frequently exhibit the immunoreactive enteroviral protein VP1 in β-cells compared to controls. This is associated with increased expression of protein kinase R, degradation of the cell survival marker MCL1, and, consequently, heightened susceptibility to apoptosis [51].

The complex interrelationships between the gut microbiota composition, the immune system, and intestinal wall permeability in T1DM pathogenesis are being intensively investigated, although several mechanisms remain unclear. Systematic reviews based on the 16S rRNA sequencing analysis have shown that T1DM patients exhibit a decreased* Firmicutes/Bacteroidetes ratio*, reduced relative abundance of Clostridium and Prevotella, along with an increased proportion of *Bacteroides* and *Ruminococcus* compared to controls.

These microbial shifts are understood to contribute to impaired intestinal permeability, chronic inflammation, and activation of autoimmune reactions, potentially facilitating the realization of the T1DM genetic risk, although causality requires further investigation [52].

For instance, a bioinformatic analysis of the B:9-23 epitope from various bacteria revealed that Parabacteroides distasonis carries peptide sequences similar to the insulin β-chain, which can potentially trigger an autoimmune response. Interestingly, T-cell clones targeting preproinsulin peptides were found to exhibit high cross-reactivity with peptides from the Bacteroides and Clostridium species [53].

Bidirectional Mendelian randomization has indicated that the presence of the phylum Bacteroidetes is associated with an increased risk of T1DM (OR = 1.24; 95% CI: 1.01–1.53; p = 0.044). Subcategories, including the class Bacteroidia and order Bacteroidales, also showed statistically significant contributions (OR = 1.28; 95% CI: 1.06–1.53; p = 0.009). In contrast, bacteria belonging to the genus Eubacterium eligens  had a protective quality (OR = 0.64; 95% CI: 0.50–0.81; p = 2.84 × 10^-4^) [54].

Furthermore, intestinal permeability is increased in T1DM patients even before the clinical onset, specifically during the preclinical stage characterized by the presence of two or more autoantibodies, without signs of impaired carbohydrate metabolism. This is likely to do with zonulin activation, a key regulator of tight junctions in the gut, whose modulation increases intestinal permeability, alongside alterations in the composition and quantity of the protective mucus layer [55, 56].

A comparative metaproteomic analysis of fecal samples from T1DM patients, individuals at the preclinical stage of T1DM, and controls revealed a link between altered gut microbiome, intestinal permeability, and the host immune system. Decreased levels of  Alistipes and Faecalibacterium prausnitzii, along with increased Bacteroidetes, was observed in both T1DM and preclinical subjects. These changes correlated with elevated levels of inflammatory proteins (galectin-3 and fibrillin-1) and increased intestinal permeability resulting from enhanced mucin degradation and reduced butyrate production [57].

Other environmental factors that potentially increase T1DM risk include absence of breastfeeding, cesarean section, early antibiotic exposure, psychological stress, and specific dietary patterns (high intake of dairy products, eggs, and root vegetables) [58].

Recent years have also highlighted the role of vitamin D deficiency, which may disrupt immune regulation and increase the risk of autoimmune diseases, including T1DM. Studies indicate that low vitamin D levels in children are associated with a higher disease risk, particularly in those with a genetic predisposition [59].

Moreover, current publications analyze the impact of urbanization, air pollution, and environmental factors on the immune status and the incidence of autoimmune diseases, including T1DM. Air pollution, especially fine particulate matter, may promote inflammatory responses and immune dysregulation [60].

The COVID-19 pandemic has drawn researchers’ attention to the role of viral and post-viral immune reactions: emerging data suggest an increase in T1DM cases among children following SARS-CoV-2 infection, although the mechanisms underlying this phenomenon are not fully understood and remain under debate [61, 62].

In summary, the role played by environmental factors in the realization of the genetic risk of T1DM is evident. However, the absence of a single specific trigger complicates the identification of at-risk groups and the development of targeted preventive strategies.

## GENETIC RISK FACTORS FOR AUTOIMMUNE DIABETES

In the general population, the lifetime risk of developing T1DM is approximately 0.4%. The contribution of genetic predisposition is estimated at 40–60%. The risk to the second monozygotic twin, if one twin is already diagnosed, is approximately 25–50%, whereas for dizygotic twins, this figure does not exceed 6%. Nearly all twins with detected autoantibodies eventually develop clinical T1DM. In a Finnish population-based cohort of 22.650 twin pairs, the cumulative incidence of T1DM among initially discordant monozygotic twins was 65%; by age 60, 78% had experienced persistent seroconversion or developed T1DM [63]. The risk is higher in the siblings of patients, reaching 5–10% by age 20, while for the children of parents with T1DM, the risk is lower: 6–9% for the children of affected fathers and 1.3–4% for the children of affected mothers [64]. Furthermore, healthy siblings sharing two identical HLA haplotypes with an affected relative are at a higher risk than those sharing one or no haplotype [65].

Early research focused on the HLA region on the short arm of chromosome 6, revealing a significant contribution of genes, alleles, and haplotypes of the major histocompatibility complex (MHC) to T1DM development [66]. An HLA haplotype is a group of HLA genes inherited together as a single Mendelian trait. The combination of two such haplotypes constitutes an individual’s HLA genotype. The most significant risk haplotypes are DRB1*03:01–DQA1*05:01–DQB1*02:01 (HLA-DR3) and DRB1*04:XX–DQA1*03:01– DQB1*03:02 (HLA-DR4-DQ8). The highest risk is to compound heterozygotes carrying both haplotypes (OR = 17). The risk is lower with homozygous combinations or a single haplotype. At least one of these haplotypes is present in 85% of T1DM patients. Conversely, some HLA class II haplotypes, such as HLA-DRB1*15:01–DQA1*01:02–DQB1*06:02 (HLA-DR15-DQ6.2), are capable of strong protective effects, reducing T1DM risk by more than 20-fold [67].

An investigation of the genomic architecture of HLA-DRB1 in the Swedish population demonstrated that changes in the amino acid residues β71, β74, and β86 significantly influence T1DM risk. These alterations modify the antigen–anchor sequences p1, p4, p7, and possibly p6. The highest risk is associated with the “lysine-alanine-glycine” motif (OR = 3.64; p = 3.19 × 10-64). Other motifs, such as “glutaminealanine-valine” (OR = 2.55; p = 0.025), “arginine-alanine-glycine” (OR = 1.93; p = 0.043), and “argininealanine-valine” (OR = 1.56; p = 0.003), are associated with a lower risk, whereas the “arginine-glutamineglycine” and “arginine-glutamine-valine” motifs demonstrate a pronounced protective capacity (OR = 0.11; p = 4.23 × 10^-4^). The presence of the “lysine-alanineglycine” motif is associated with increased frequency of IA-2A autoantibodies (OR = 2.13) but a lower frequency of GADA (OR = 0.83) [68]. Although the prevalence of HLA-DR3 and HLA-DR4-DQ8 in Caucasian populations reaches 4.5% and 12.5%, respectively, T1DM develops in less than 1% of carriers of these haplotypes [69] (Table 4).

Subsequent studies of candidate genes have identified other loci associated with the risk of developing T1DM. A key example is the INS gene. The INS gene possesses a variable number of tandem repeats (VNTR) in its 5’-untranslated region. Class I VNTR alleles (26–63 repeats) are associated with an increased risk of T1DM (OR > 2), whereas class III VNTR alleles (140–210 repeats) exert a protective effect [65]. The efficiency of INS intron 1 splicing influences insulin expression in the thymus, thereby regulating the selection of insulin-specific T cells [77]. Furthermore, mutations in the INS gene are a known cause of neonatal diabetes, leading to impaired preproinsulin maturation and pancreatic β-cell death [78].

Lymphoid-specific phosphatase (LYP), encoded by the PTPN22 gene (chromosome 1p13), is involved in the regulation of the T-cell activity. The functional polymorphism rs2476601 (c.1858C > T, p.R620W), prevalent in Northern Europe, boosts tyrosine kinase activity and alters the composition of the regulatory T-cell (Treg) population. Although this might seem counterintuitive given the suppressive activity of Tregs, it is believed to reflect a more complex immune response regulation [79].

**Table 4:** The prevalence of risk and protective HLA haplotypes in various ethnic groups in the Russian Federation

Ethnicity	High-risk HLA loci and haplotypes	OR	Frequency in T1D patients, %	Protective HLA loci and genotypes	Frequency in controls, %	OR
Russian (Moscow) [70]	DRB1*04-DQA1*03:01-DQB1*03:04	4.0	0.17	DRB1*11-DQA1*05:01-DQB1*03:01	12.5	-
	DRB1*04-DQA1*03:01-DQB1*03:02	5.99	8.5	DRB1*13-DQA1*01:02-DQB1*06:02/8	8.5	-
	DRB1*017(3)-DQA1*05:01-DQB1*02:01	4.1	10	DRB1*07-DQA1*02:01-DQB1*02:01	≈15.5	-
Russian (Vologda) [70]	DRB1*04-DQA1*03:01-DQB1*03:04	9.22	-	DRB1*11-DQA1*05:01-DQB1*03:01	9.1	-
	DRB1*04-DQA1*03:01-DQB1*03:02	4.26	11.6	DRB1*13-DQA1*01:02-DQB1*06:02/8	11.1	-
	DRB1*017(3)-DQA1*05:01-DQB1*02:01	4.21	7.4	DRB1*07-DQA*02:01-DQB1*02:01	≈11	-
Nenets [70]	DRB1*04-DQA1*03:01=DQB1*03:02	-	11.5	DRB1*11-DQA1*05:01-DQB1*03:01	32.8	-
	DRB1*017(3)-DQA1*05:01-DQB1*02:02	-	1.6	DRB1*13-DQA1*01:02-DQB1*06:02/8	16.4	-
	DRB1*01-DQA1*01:01-DQB1*05:01	-	3.3	DRB1*07-DQA1*02:01-DQB1*02:01	≈25	-
Udmurts [71]	DRB1*04-DQA1*03:01-DQB1*03:02	12	2.6	DRB1*07-DQA*02:01-DQB1*02:01	24.23	0.36
	DRB1*017(3)-DQA1*05:01-DQB1*02:02	4.9	3.6	DRB1*07-DQA*02:01-DQB1*03:03	8.76	≤0.18
	DQA1*03:01-DQB1*03:02 or/and *02:01	14.2	62			
Tuvans [72]	DRB1*03-DQAl*05:01-DQB1*02:01	6.3	26.7	-	-	-
Bashkirs [73]	DRB1*04-DQB1*03:02	8.82	28	DRB1*15-DQB1*06:02/8	26.3	0.04
	DRB1*17-DQB1*02:01	6.47	56	DRB1*11-DQB1*03:01	14	0.18
	DQA1*03:01-DQB1*03:02	10.62	32			
Buryats [74]	DRB1*08-DQA1*03:01-DQB1*03:02	7.83	6.1	DRB1*15-DQA1*01:02-DQB1*06:02/8	9.8	0.25
	DRB1*04-DQA1*03:01-DQB1*03:02	5.78	8.9	DRB1*11-DQA1*05:01-DQB1*03:01	4.7	0.35
Yakuts [75]	DRB1*017(3)	8.47	30.4	DRB1*11	8.8	0.1
	DRB1*04	4.27	46.1	DQB1*06:02/8	19.6	0.17
Tatars [76]	DQB1*03:02	4.04	52	DRB1*15	28.6	0.03
	DRB1*017(3)	2.42	51.4	DQB1*06:02/8	31	0.11

The cytotoxic T-lymphocyte-associated antigen-4 (CTLA-4) gene, located on chromosome 2q33, encodes a co-receptor that suppresses cytotoxic T-cell activation and interleukin-2 (IL-2) production. The polymorphic variants rs3087243 and rs231775 are associated with an increased risk of T1DM (OR = 1.31 and 1.47, respectively) [26]. In cancer therapy, immune checkpoint inhibitors blocking the CTLA-4 and PD-1/PD-L1 pathways are used and can sometimes trigger autoimmune reactions, including diabetes that resembles T1DM. An analysis of 91 such diabetes cases revealed that most patients (71%) received anti-PD-1 therapy, and that the dominant HLA haplotypes were DR4, DR3, DR9, and A2 [80].

The UBASH3A gene (also known as STS-2, TULA, and CLIP4), located in the 21q22.3 locus on human chromosome 21, is expressed predominantly in T cells and encodes a ubiquitin-associated protein containing the SH3 domain (UBASH3A) [81]. UBASH3A inhibits T-cell receptor (TCR)-induced signaling to nuclear factor-kB (NF-kB), leading to suppressed IL-2 expression. Additionally, UBASH3A regulates the synthesis and dynamics of the CD3/TCR coreceptor complex, further attenuating signal transduction and reducing IL-2 production. The minor alleles of polymorphisms rs80054410 (g.43836010T>C) and rs11203203 (g.43836186G>A) enhance UBASH3A expression in human naïve T cells, which is associated with T1DM risk [82]. A statistically significant additive effect on the T1DM risk (p = 0.029) was also identified in the concurrent presence of SNP rs11203203 in UBASH3A and SNP rs2476601 in PTPN22 [83].

Substantial contributions to T1DM development are also made by the interleukin-2 receptor subunit alpha (IL2RA) gene, cytokine genes (primarily  IL-4 and IL-13), and the  interferon-induced helicase IFIH1 gene [4]. The IL2RA gene comprises eight exons and encodes the alpha chain of the IL-2 receptor, a potent lymphocyte growth factor. IL2RA expression in regulatory T cells (CD4^+^CD25+) is essential for immune response control and for preventing autoimmunity. Studies have shown that the rs52580101 polymorphism, located in intron 1 of IL2RA, is the most prevalent among T1DM patients [84].

A study conducted in Kuwait demonstrated that the IL-4 (CC) and IL-13 (AA/Q) genotypes come with risk. Co-inheritance of the IL-4 CC genotype (−590C/T, rs2243250) and the IL-13 AA/Q genotype polymorphism p.(Arg130Glu) with the high-risk HLA genotypes (HLA-DQ and HLA-DR) was also identified. These findings advance our understanding of the genetic predisposition to T1DM in Kuwaiti children [85]. The data are consistent with the results from Saudi Arabia, where young T1DM patients (age of onset ≤13 years) carrying high-risk HLA variants also showed an increased frequency of IL-4 rs2070874 (C/C) SNP [86].

The cytosolic viral RNA sensor IFIH1 (also known as MDA5) is a gene that significantly affects T1DM risk and plays a key role in antiviral immunity. A non-synonymous polymorphism – rs1990760 – in this gene results in an alanine-to-threonine substitution at position 946 [87]. Given the role played by IFIH1 in antiviral immunity, attempts have been made to look for enteroviral RNA in patients with autoantibodies or recently diagnosed T1DM. Although the studies remain limited by sample size, they are an important contribution to our understanding of the role of viruses as triggers of the autoimmune process [88].

The product of the BACH_2_ gene accelerates the cytokine-induced apoptosis of pancreatic β-cells via the activation of the JNK1/BIM signaling pathway, whereas BACH_2_ overexpression exerts the opposite effect. SNPs rs375724724 and rs11755527 are reported to be associated with T1DM risk in Caucasians, and SNP rs3757247 is associated with risk in the Japanese population [89]. Conversely, SNP rs11755527 showed no association with T1DM in a Southern Brazilian population of mixed ethnic ancestry, highlighting potential ethnic differences in the prevalence and significance of this variant [90] (Table 5).

**Table 5:** Significant non-HLA genetic associations with T1DM risk^*^

Gen	SNP	Risk allele	OR	95% CI	p-value
INS	rs689	T	2.21	[2.08-2.34]	1 × 10^-160^
PTPN22	rs2476601	T	1.98	[1.82-2.15]	2 × 10^-80^
CTLA-4	rs926169	T	1.28	[1.15-1.42]	9 × 10^-6^
UBASH3A	rs11203203	A	1.14	[1.1-1.17]	3 × 10^-17^
IFIH1	rs1990760	A	1.18	[1.11-1.23]	2 × 10^-11^
BACH_2_	rs11755527	G	1.13	[1.08-1.19]	5 × 10^-12^

^*^Data source: GWAS Catalog - https://www.ebi.ac.uk/gwas/home

Given the accumulated data on the multiple genetic variants associated with T1DM and the absence of clear inheritance patterns, it has been proposed that disease development be considered to result from the additive interaction of genes, or epistasis [91]. Consequently, in 2014, a multifactorial logistic regression model, GRS-1, was developed, incorporating high-risk HLA variants and 40 additional SNPs. The efficacy of the model was evaluated using the data from the German longitudinal studies “BABYDIAB” and “BABYDIET.” Use of HLA variants alone yielded an area under the ROC curve (AUC) of 0.82 (95% CI: 0.80–0.83), while the inclusion of additional SNPs increased the AUC to 0.87 (95% CI: 0.86–0.88) [92].

The ongoing study “The Global Platform for the Prevention of Autoimmune Diabetes-02” (GPPAD-02) aims to assess the possibility of primary T1DM prevention through daily oral administration of fixed insulin doses. The concept involves stimulating antigen uptake for presentation to the immune system to maintain tolerance. The inclusion criterion is high genetic risk, assessed either by 46 SNPs or by three SNPs if a first-degree relative has T1DM. Screening of 50,669 infants conducted from October 2017 to December 2018 identified high genetic risk in 1.1% of those examined, corresponding to an approximately 10% probability of developing multiple β-cell autoantibodies by age 6 [93].

With the expanding list of identified SNPs, a new genetic model, GRS-2, incorporating 67 SNPs, was proposed. It demonstrated a statistically significant improvement in risk prediction (the AUC increased from 0.893 to 0.92; p <  0.0001) according to the UK Biobank data [94]. Further modification of the model (GRS-2’), by including SNPs from the HLA-DQ locus (rs9273363) and non-HLA loci (rs926169, rs10788599, and rs56380902), improved risk prediction in both the European and African American populations [95]. Notably, a model specifically designed for African Americans showed better performance (AUC 0.871) than the European model, underscoring the necessity of using population-specific values for T1DM risk assessment [96].

## EPIGENETIC MODIFICATIONS

Discordance in genetically associated diseases among monozygotic twins may be due to epigenetic modifications formed through the interaction of the genotype with the environment. These changes result in a unique phenotype. Monozygotic twins are epigenetically indistinguishable during early life; however, differences in the overall content and distribution of DNA 5-methylcytosine and histone acetylation are observed in older age groups, consistent with the concept of epigenetic drift [97].

Epigenetic regulation involves modifications of gene expression that do not alter the nucleotide sequence but are heritable. The main epigenetic mechanisms include DNA methylation, post-translational histone modifications, and RNA interference.

For a DNA segment to be used as a template, the promoter and other gene regulatory regions, including enhancers, must be accessible to transcription factors and other regulatory complexes. DNA methylation reduces chromatin accessibility in regulatory regions, which can impair the binding of transcription factors and consequently alter gene expression [98]. Methylation is a form of covalent DNA modification that does not change its sequence and involves the transfer of a methyl group (CH_3_ ) from S-adenosylmethionine to the 5-position of the cytosine pyrimidine ring [99]. The effects of methylation depend on its location: methylation within the gene’s body is associated with reduced transcriptional processivity of RNA polymerase, while methylation in the promoter or first intron is linked to impaired transcription initiation [100].

Several studies have identified specific DNA methylation profiles in T1DM genetic risk regions, such as the INS gene promoter (SNP rs689), IL2RA, tumor necrosis factor-alpha (TNFα), and HLA class II haplotypes [101, 102, 103]. A limitation of the early studies was that methylation levels were measured only after disease onset, since changes could have been caused by chronic hyperglycemia, active autoimmune processes, and medication. However, the “DAISY” case-control study identified DNA hypermethylation preceding seroconversion, confirming the significant contribution of epigenetic changes to the realization of genetic T1DM risk [104].

Reduced FoxP3 gene expression is associated with the development of several autoimmune diseases, including T1DM. DNA methylation in CD4^+^ T cells from patients with LADA was found to be significantly increased compared to controls. The FoxP3 promoter region was also hypermethylated in CD4^+^ T cells, accompanied by decreased FoxP3 mRNA expression levels (normalized to β-actin), as measured by realtime PCR [105].

Histones undergo post-translational modifications of specific amino acid residues in the N-terminal region. These modifications regulate the chromatin structure, significantly influencing gene transcription, DNA repair, and chromosome condensation. One significant study revealed increased methylation of lysine 9 on histone H3 (H3K9me2) at the CTLA-4 gene promoter and simultaneously decreased H3K9me2 at the ICOS gene promoter in T1DM patients, which was associated with the development of T-cell autoimmunity [106]. Monocytes from T1DM patients had lower levels of H3K9Ac (4 kb upstream of HLA-DRB1) and higher levels of H3K9Ac (4 kb upstream of HLA-DQB1) [3]. Increased acetylation at these sites correlated with enhanced transcription in a monocyte cell line. However, at the time of the study, it was impossible to determine whether these differences were a cause of the disease or a consequence of chronic hyperglycemia. Yanfei Wang et al. identified increased levels of histone H3 acetylation (H3AC) in T-lymphocytes from T1DM patients using Western blotting. A subgroup analysis showed no significant difference in global acetylation levels between compensated and uncompensated patients. A positive correlation was also established between acetylation levels in the ICOS promoter region (−137/−55) and ICOS mRNA expression (r = 0.655, p = 0.021). Bivariate correlation between ICOS mRNA expression and clinical parameters (glucose level, glycated hemoglobin, C-peptide, GADA titer, and blood pressure) confirmed a positive association between GADA titer and ICOS mRNA expression [107].

**Fig. 2:**
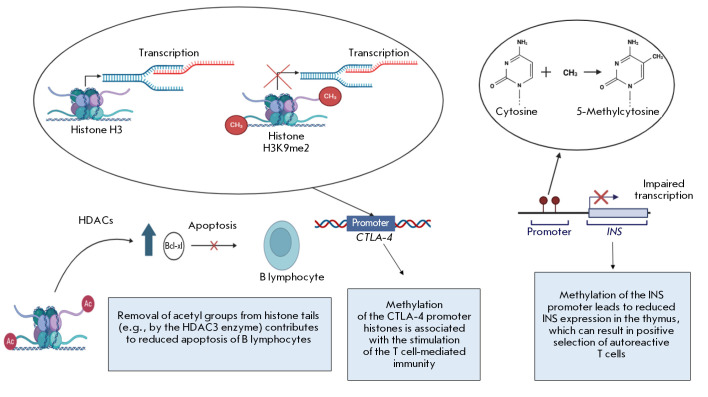
Examples of the epigenetic markers of T1DM according to the studies by Hu Qibo et al. [109], Miao Feng et al. [106], and Pahkuri Sirpa et al. [102]. The authors created the original figure using BioRender

Histone deacetylases (HDACs) catalyze the removal of acetyl groups from histone tails using coenzyme A. Based on intracellular localization, HDACs are divided into three classes: Class I – HDAC 1, 2, 3, and 8 (exclusively nuclear); Classes IIa and IIb – HDAC 4, 5, 6, 7, 9, and 10 (predominantly cytoplasmic); and Class III – sirtuin (SIRT) enzymes 1–7 (localized in the nucleus, cytoplasm, or mitochondria) [108]. For example, HDAC3 can upregulate the Bcl-xl protein (a key regulator of the mitochondrial apoptosis pathway) by suppressing microRNA-296-5p expression, leading to inhibited apoptosis of B lymphocytes, which are directly involved in the autoimmune response against pancreatic β-cells [109]. The scientific community is actively discussing the potential therapeutic use of HDAC inhibitors (HDACi). For instance, the polyaminobenzamide pan-HDACi THS-78-5 protects against IL-1β-induced β-cell death by attenuating inducible nitric oxide synthase expression and NF-κB transactivation, and the highly selective HDAC3 inhibitor BRD3308 exhibited protective properties in vitro and in vivo [110] (Fig. 2).

MicroRNAs control gene expression through influence on mRNA stability and translation through direct inhibition and degradation. Epigenetic changes in microRNAs affect the cell cycle and immune response [111]. For example, microRNA-21 (miR-21), whose overexpression impairs β-cell development in animal models, increases caspase-3 levels and accelerates β-cell apoptosis by targeting Bcl-2 gene translation [112]. MicroRNA-146a (miR-146a) reduces the pro-inflammatory response by suppressing the TRAF6 and IRAK1 genes. Decreased miR-146a expression has been noted in T1DM patients; in animal models, increased TRAF6 expression was found to worsen glucose-induced endothelial damage. Reduced miR-146a expression is also associated with increased GADA antibody titers [113].

## CONCLUSIONS

The clinical variability of type 1 diabetes (T1DM) is due to its significant genetic heterogeneity and the presence of epistatic interactions between target genes. The loci associated with T1DM exhibit substantial differences in frequency and penetrance, with variations observed even in neighboring regions, not to mention different ethnic groups.

Identifying the specific trigger factors contributing to seroconversion remains a subject of active scientific research. However, given the significant global increase in T1DM incidence, the important role of epigenetic modifications in actuating genetic risk becomes evident. Epigenetic changes may serve as the link between genetic predisposition and external environmental exposures, explaining the variability in disease course and age of onset.

In this context, the identification of new candidate genes, the creation and refinement of logistic regression risk assessment models, and in-depth study of epigenetic mechanisms open broad prospects for researchers, not only for early diagnosis and potential prevention of T1DM, but also for developing fundamentally new therapeutic approaches. Utilizing the latest spatial genomics methods and integrating genetic and epigenetic data will allow for a more detailed study of the complex intercellular and intracellular interactions shaping the pathological process.

Thus, further development of genomic and epigenomic analysis techniques, along with the implementation of personalized medicine approaches, will contribute significantly to our understanding of T1DM pathogenesis and to the creation of effective methods for the prevention and treatment of this autoimmune disease.

## Abbreviations

T1DM: type 1 diabetes mellitus
T2DM: type 2 diabetes mellitus
DM: diabetes mellitus
GWAS: genome-wide association study
NGS: next-generation sequencing
ICA: islet cell antibody
IAA: insulin autoantibody
IA-2: islet antigen 2
GADA: glutamic acid decarboxylase antibody
ZnT8A: zinc transporter 8 protein autoantibody
LADA: latent autoimmune diabetes in adults
LADY: latent autoimmune diabetes in young
IDS: immunology of diabetes society
MODY: maturity-onset diabetes of the young
MIDD: maternally inherited diabetes and deafness
SNP: single-nucleotide polymorphism
CI: confidence interval
OS: odds ratio

## References

[1] Dedov II., Shestakova MV., Vikulova OK. (2023). Diabetes mellitus in the Russian Federation: dynamics of epidemiological indicators according to the Federal Register of Diabetes Mellitus for the period 2010–2022.. Diabetes Mellit..

[2] (2025). Federal Register of Diabetes Mellitus. Endocrinology Research Centre. Published 2024. Accessed March 24, 2025.. Clinical and epidemiological monitoring of diabetes mellitus..

[3] Akil AA., Jerman LF., Yassin E., Padmajeya SS., Al-Kurbi A., Fakhro KA. (2020). Reading between the (genetic) lines: how epigenetics is unlocking novel therapies for type 1 diabetes.. Cells..

[4] Noble JA. (2024). Fifty years of HLA-associated type 1 diabetes risk: history, current knowledge, and future directions.. Front Immunol..

[5] Erlich H., Valdes AM., Noble J. (2008). HLA DR-DQ haplotypes and genotypes and type 1 diabetes risk: analysis of the type 1 diabetes genetics consortium families.. Diabetes..

[6] Chiou J., Geusz RJ., Okino ML. (2021). Interpreting type 1 diabetes risk with genetics and single-cell epigenomics.. Nature.

[7] Zhao LP., Alshiekh S., Zhao M. (2016). Next-generation sequencing reveals that HLA-DRB3, -DRB4, and -DRB5 may be associated with islet autoantibodies and risk for childhood type 1 diabetes.. Diabetes..

[8] Lezzi M., Aloi C., Salina A. (2022). Diabetes mellitus diagnosed in childhood and adolescence with negative autoimmunity: results of genetic investigation.. Front Endocrinol (Lausanne)..

[9] (2022). American Diabetes Association Professional Practice Committee. Classification and diagnosis of diabetes:. Standards of medical Care in Diabetes-2022..

[10] (1986). Eisenbarth GS. Type I diabetes mellitus. A chronic autoimmune disease.. N Engl J Med..

[11] (2021). Korneva KG, Strongin LG, Zagainov VE. Immunological predictors of type 1 diabetes mellitus.. Diabetes Mellit..

[12] Zajec A., Trebušak Podkrajšek K., Tesovnik T. (2022). Pathogenesis of type 1 diabetes: established facts and new insights.. Genes (Basel)..

[13] Barcenilla H., Åkerman L., Pihl M., Ludvigsson J., Casas R. (2019). Mass cytometry identifies distinct subsets of regulatory T cells and natural killer cells associated with high risk for type 1 diabetes.. Front Immunol..

[14] Nikonova TV., Apanovich PV., Pekareva EV. (2010). The role of regulatory CD4+CD25+high T-lymphocytes and their functional activity in the development of type 1 diabetes mellitus.. Diabetes Mellit..

[15] Afyouni H., Haris B., Syed N. (2022). Sib-pair subgroup familial type 1 diabetes mellitus in children in the state of Qatar.. PLoS One..

[16] Hu J., Zhang R., Zou H., Xie L., Zhou Z., Xiao Y. (2022). Latent autoimmune diabetes in adults (LADA): from immunopathogenesis to immunotherapy.. Front Endocrinol (Lausanne)..

[17] Mishra R., Chesi A., Cousminer DL. (2017). Relative contribution of type 1 and type 2 diabetes loci to the genetic etiology of adult-onset, non-insulin-requiring autoimmune diabetes.. BMC Med..

[18] Cousminer DL., Ahlqvist E., Mishra R. (2018). First genome-wide association study of latent autoimmune diabetes in adults reveals novel insights linking immune and metabolic diabetes.. Diabetes Care..

[19] Dong F., Yang G., Pan HW. (2014). The association of PTPN22 rs2476601 polymorphism and CTLA-4 rs231775 polymorphism with LADA risks: a systematic review and meta-analysis.. Acta Diabetol..

[20] Nan X., Li X., Xiang Y. (2022). Thyroid autoantibody distribution in patients with latent autoimmune diabetes in youth: a multicenter, national survey.. Ann Transl Med..

[21] Peng Y., Li X., Xiang Y. (2022). GAD65 antibody epitopes and genetic background in latent autoimmune diabetes in youth (LADY).. Front Immunol..

[22] Sun Q., Yang M., Jing Y. (2025). Latent autoimmune diabetes in youth.. Frontiers in Immunol..

[23] Del Chierico F., Rapini N., Deodati A., Matteoli MC., Cianfarani S., Putignani L. (2022). Pathophysiology of type 1 diabetes and gut microbiota role.. Int J Mol Sci..

[24] Patterson CC., Harjutsalo V., Rosenbauer J. (2019). Trends and cyclical variation in the incidence of childhood type 1 diabetes in 26 European centres in the 25 year period 1989–2013: a multicentre prospective registration study.. Diabetologia..

[25] Gong B., Yang W., Xing Y., Lai Y., Shan Z. (2025). Global, regional, and national burden of type 1 diabetes in adolescents and young adults.. Pediatr Res..

[26] (2024). Centers for Disease Control and Prevention.. National Diabetes Statistics Report..

[27] Wagenknecht LE., Lawrence JM., Isom S. (2023). Trends in incidence of youth-onset type 1 and type 2 diabetes in the USA, 2002–18: results from the population-based SEARCH for Diabetes in Youth study.. Lancet Diabetes Endocrinol..

[28] Ogrotis I., Koufakis T., Kotsa K. (2023). Changes in the global epidemiology of type 1 diabetes in an evolving landscape of environmental factors: causes, challenges, and opportunities.. Medicina (Kaunas)..

[29] Rogers MAM., Kim C., Banerjee T., Lee JM. (2017). Fluctuations in the incidence of type 1 diabetes in the United States from 2001 to 2015: a longitudinal study.. BMC Med..

[30] Tönnies T., Brinks R., Isom S. (2023). Projections of type 1 and type 2 diabetes burden in the U.S. population aged < 20 years through 2060: the SEARCH for diabetes in youth study.. Diabetes Care..

[31] Unnikrishnan R., Anjana RM., Mohan V. (2016). Diabetes mellitus and its complications in India.. Nat Rev Endocrinol..

[32] Weng J., Zhou Z., Guo L. (2018). Incidence of type 1 diabetes in China, 2010-13: population based study.. BMJ..

[33] Li GH., Huang K., Dong GP. (2022). Clinical incidence and characteristics of newly diagnosed type 1 diabetes in Chinese children and adolescents: a nationwide registry study of 34 medical centers.. Front Pediatr..

[34] de Almeida-Pititto B., Eliaschewitz FG., de Paula MA., Ferreira GC. (2022). BrazIliaN type 1 & 2 diabetEs disease registry (BINDER): longitudinal, real-world study of diabetes mellitus control in Brazil.. Front Clin Diabetes Healthc..

[35] Negrato CA., Lauris JRP., Saggioro IB. (2017). Increasing incidence of type 1 diabetes between 1986 and 2015 in Bauru, Brazil.. Diabetes Res Clin Pract..

[36] Gomes MB., Porto LC., Silva DA. (2022). HLA genotypes and type 1 diabetes and its relationship to reported race/skin color in their relatives: a Brazilian multicenter study.. Genes (Basel)..

[37] (2025). National Paediatric Diabetes Audit (NPDA) annual reports.. Royal College of Paediatrics and Child Health..

[38] (2025). National Diabetes Audit (NDA) 2024-25 quarterly report for England, Integrated Care Board (ICB), Primary Care Network (PCN) and GP practice.. NHS England..

[39] Buchmann M., Tuncer O., Auzanneau M. (2023). Incidence, prevalence and care of type 1 diabetes in children and adolescents in Germany: time trends and regional socioeconomic situation.. J Health Monit..

[40] Scheuing N., Wiegand S., Bächle C. (2015). Impact of maternal country of birth on type-1-diabetes therapy and outcome in 27,643 children and adolescents from the DPV registry.. PLoS One..

[41] Neu A., Willasch A., Ehehalt S., Kehrer M., Hub R., Ranke MB. (2001). Diabetes incidence in children of different nationalities: an epidemiological approach to the pathogenesis of diabetes.. Diabetologia..

[42] Newhook LA., Penney S., Fiander J., Dowden J. (2012). Recent incidence of type 1 diabetes mellitus in children 0-14 years in Newfoundland and Labrador, Canada climbs to over 45/100,000: a retrospective time trend study.. BMC Res Notes..

[43] Aronson R., Brown RE., Abitbol A. (2021). The Canadian LMC diabetes registry: a profile of the demographics, management, and outcomes of individuals with type 1 diabetes.. Diabetes Technol Ther..

[44] Zayed H. (2016). Genetic epidemiology of type 1 diabetes in the 22 Arab countries.. Curr Diab Rep..

[45] Alotaibi A., Perry L., Gholizadeh L., Al-Ganmi A. (2017). Incidence and prevalence rates of diabetes mellitus in Saudi Arabia: an overview.. J Epidemiol Glob Health..

[46] Wu R., Mumtaz M., Maxwell AJ. (2023). Respiratory infections and type 1 diabetes: potential roles in pathogenesis.. Rev Med Virol..

[47] Faulkner CL., Luo YX., Isaacs S., Rawlinson WD., Craig ME., Kim KW. (2021). The virome in early life and childhood and development of islet autoimmunity and type 1 diabetes: a systematic review and meta-analysis of observational studies.. Rev Med Virol..

[48] Lemos JRN., Hirani K., von Herrath M. (2024). Immunological and virological triggers of type 1 diabetes: insights and implications.. Front Immunol..

[49] Ruiz PLD., Tapia G., Bakken IJ. (2018). Pandemic influenza and subsequent risk of type 1 diabetes: a nationwide cohort study.. Diabetologia..

[50] Op de Beeck A., Eizirik DL. (2016). Viral infections in type 1 diabetes mellitus--why the β cells?. Nat Rev Endocrinol..

[51] Richardson SJ., Leete P., Bone AJ., Foulis AK., Morgan NG. (2013). Expression of the enteroviral capsid protein VP1 in the islet cells of patients with type 1 diabetes is associated with induction of protein kinase R and downregulation of Mcl-1.. Diabetologia..

[52] Zhou H., Zhao X., Sun L. (2020). Gut microbiota profile in patients with type 1 diabetes based on 16S rRNA gene sequencing: a systematic review.. Dis Markers..

[53] Forouhi NG., Wareham NJ. (2014). Epidemiology of diabetes.. Medicine (Abingdon)..

[54] Luo M., Sun M., Wang T. (2023). Gut microbiota and type 1 diabetes: a two-sample bidirectional Mendelian randomization study.. Front Cell Infect Microbiol..

[55] Wood Heickman LK., DeBoer MD., Fasano A. (2020). Zonulin as a potential putative biomarker of risk for shared type 1 diabetes and celiac disease autoimmunity.. Diabetes Metab Res Rev..

[56] Peters A., Wekerle H. (2019). Autoimmune diabetes mellitus and the leaky gut.. Proc Natl Acad Sci U S A..

[57] Gavin PG., Mullaney JA., Loo D. (2018). Intestinal metaproteomics reveals host-microbiota interactions in subjects at risk for type 1 diabetes.. Diabetes Care..

[58] Stene LC., Norris JM., Rewers MJ. (2023). Risk factors for type 1 diabetes. In: Lawrence JM, Casagrande SS, Herman WH, et al, eds. Diabetes in America. National Institute of Diabetes and Digestive and Kidney Diseases (NIDDK);. ???.

[59] Dong JY., Zhang WG., Chen JJ., Zhang ZL., Han SF., Qin LQ. (2013). Vitamin D intake and risk of type 1 diabetes: a meta-analysis of observational studies.. Nutrients..

[60] Rewers M., Ludvigsson J. (2016). Environmental risk factors for type 1 diabetes.. Lancet..

[61] Kamrath C., Mönkemöller K., Biester T. (2020). Ketoacidosis in children and adolescents with newly diagnosed type 1 diabetes during the COVID-19 pandemic in Germany.. JAMA..

[62] Barrett CE., Koyama AK., Alvarez P. (2022). Risk for newly diagnosed diabetes > 30 days after SARS-CoV-2 infection among persons aged < 18 years — United States, March 1, 2020–June 28, 2021.. MMWR Morb Mortal Wkly Rep..

[63] Redondo MJ., Jeffrey J., Fain PR., Eisenbarth GS., Orban T. (2008). Concordance for islet autoimmunity among monozygotic twins.. N Engl J Med..

[64] Lee HS., Hwang JS. (2019). Genetic aspects of type 1 diabetes.. Ann Pediatr Endocrinol Metab..

[65] Steck AK., Rewers MJ. (2011). Genetics of type 1 diabetes.. Clin Chem..

[66] Primavera M., Giannini C., Chiarelli F. (2020). Prediction and prevention of type 1 diabetes.. Front Endocrinol (Lausanne)..

[67] Sharp SA., Weedon MN., Hagopian WA., Oram RA. (2018). Clinical and research uses of genetic risk scores in type 1 diabetes.. Curr Opin Genet Dev..

[68] Zhao LP., Papadopoulos GK., Lybrand TP. (2021). The KAG motif of HLA-DRB1 (β71, β74, β86) predicts seroconversion and development of type 1 diabetes.. EBioMedicine..

[69] Mameli C., Triolo TM., Chiarelli F., Rewers M., Zuccotti G., Simmons KM. (2023). Lessons and gaps in the prediction and prevention of type 1 diabetes.. Pharmacol Res..

[70] Kuraeva TL., Zubov LA., Titovich EV. (2017). HLA-haplotypes and the risk of developing diabetes of type 1 diabetes in the native population of the Nenets Autonomous district.. Diabetes Mellit..

[71] Ivanova ON., Prokof’ev SA., Zvereva YS., Kovalenko TV., Blinov AV., Peterkova VA. (2009). Class II HLA diabetogenic markers in an Udmurtian population: genotype dependence, the role of DQ trans-heterodimers.. Diabetes Mellit..

[72] Osokina IV., Boldyreva MN., Shirshina RK. (2001). HLA-markery sakharnogo diabeta 1 tipa v Tuvinskoy populyatsii.. Diabetes Mellit..

[73] Suntsov YI., Dedov II., Maslova OV. (2006). Risk razvitiya sakharnogo diabeta 1 tipav populyatsii Bashkir(po dannym HLA-genotipirovaniya).. Diabetes Mellit..

[74] Dedov II., Kolesnikova LI., Ivanova ON. (2006). Polimorfizm genov HLA klassa II i CTLA4 zdorovykh buryat i bol’nykh sakharnym diabetom 1 tipa v Buryatskoy Respublike.. Diabetes Mellit..

[75] Titovich EV., Kuraeva TL., Danilova GI. (2009). Association of type 1 diabetes mellitus (DM1) with polymorphous alleles of class II HLA genes in Yakutian and Russian populations.. Diabetes Mellit..

[76] Avzaletdinova S., Morugova TV., Mustafina OE. (2012). Allele variants of HLA II genes DRB1 and DQB1 regarding risk for type 1 diabetes mellitus in population of Bashkortostan.. Diabetes Mellit..

[77] Kralovicova J., Vorechovsky I. (2010). Allele-specific recognition of the 3′ splice site of INS intron 1.. Hum Genet..

[78] Tikhonovich YV., Petryaykina EE., Timofeev AV. (2021). Clinical, hormonal and molecular-genetic characteristics of monogenic diabetes mellitus associated with the mutations in the INS gene.. Diabetes Mellit..

[79] Valta M., Gazali AM., Viisanen T. (2020). Type 1 diabetes linked PTPN22 gene polymorphism is associated with the frequency of circulating regulatory T cells.. Eur J Immunol..

[80] de Filette JMK., Pen JJ., Decoster L. (2019). Immune checkpoint inhibitors and type 1 diabetes mellitus: a case report and systematic review.. Eur J Endocrinol..

[81] Ge Y., Paisie TK., Newman JRB., McIntyre LM., Concannon P. (2017). UBASH3A mediates risk for type 1 diabetes through inhibition of T-cell receptor–induced NF-κB signaling.. Diabetes..

[82] Ge Y., Concannon P. (2018). Molecular-genetic characterization of common, noncoding UBASH3A variants associated with type 1 diabetes.. Eur J Hum Genet..

[83] Newman JRB., Concannon P., Ge Y. (2023). UBASH3A interacts with PTPN22 to regulate IL2 expression and risk for type 1 diabetes.. Int J Mol Sci..

[84] Ranjouri MR., Aob P., Mansoori Derakhshan S., Shekari Khaniani M., Chiti H., Ramazani A. (2016). Association study of IL2RA and CTLA4 gene variants with type I diabetes mellitus in children in the northwest of Iran.. Bioimpacts..

[85] Haider MZ., Al Rushood M., Alsharhan H., Rasoul MA., Al-Mahdi M., Al-Kandari H. (2023). Association of interleukin-4, interleukin-13 gene polymorphisms, HLA-DQ and DR genotypes with genetic susceptibility of type-1 Diabetes Mellitus in Kuwaiti children.. Front Pediatr..

[86] Osman AE., Brema I., AlQurashi A., Al-Jurayyan A., Bradley B., Hamza MA. (2022). Single nucleotide polymorphism rs 2070874 at Interleukin-4 is associated with increased risk of type 1 diabetes mellitus independently of human leukocyte antigens.. Int J Immunopathol Pharmacol..

[87] Stock AJ., Gonzalez Paredes P., de Almeida LP. (2024). The IFIH1-A946T risk variant promotes diabetes in a sex-dependent manner.. Front Immunol..

[88] Sioofy-Khojine AB., Richardson SJ., Locke JM. (2022). Detection of enterovirus RNA in peripheral blood mononuclear cells correlates with the presence of the predisposing allele of the type 1 diabetes risk gene IFIH1 and with disease stage.. Diabetologia..

[89] Onuma H., Kawamura R., Tabara Y. (2019). Variants in the BACH2 and CLEC16A gene might be associated with susceptibility to insulin-triggered type 1 diabetes.. J Diabetes Investig..

[90] Dieter C., Lemos NE., Dorfman LE., Duarte GCK., Assmann TS., Crispim D. (2020). The rs11755527 polymorphism in the BACH2 gene and type 1 diabetes mellitus: case control study in a Brazilian population.. Arch Endocrinol Metab..

[91] Pang H., Lin J., Luo S. (2022). The missing heritability in type 1 diabetes.. Diabetes Obes Metab..

[92] Winkler C., Krumsiek J., Buettner F. (2014). Feature ranking of type 1 diabetes susceptibility genes improves prediction of type 1 diabetes.. Diabetologia..

[93] Winkler C., Haupt F., Heigermoser M. (2019). Identification of infants with increased type 1 diabetes genetic risk for enrollment into Primary Prevention Trials-GPPAD-02 study design and first results.. Pediatr Diabetes..

[94] Sharp SA., Rich SS., Wood AR. (2019). Development and standardization of an improved type 1 diabetes genetic risk score for use in newborn screening and incident diagnosis.. Diabetes Care..

[95] Qu HQ., Qu J., Glessner J. (2022). Improved genetic risk scoring algorithm for type 1 diabetes prediction.. Pediatr Diabetes..

[96] Onengut-Gumuscu S., Chen WM., Robertson CC. (2019). Type 1 diabetes risk in African-ancestry participants and utility of an ancestry-specific genetic risk score.. Diabetes Care..

[97] Morris BJ., Willcox BJ., Donlon TA. (2019). Genetic and epigenetic regulation of human aging and longevity.. Biochim Biophys Acta Mol Basis Dis..

[98] Bansal A., Pinney SE. (2017). DNA methylation and its role in the pathogenesis of diabetes.. Pediatr Diabetes..

[99] Minniakhmetov I., Yalaev B., Khusainova R. (2024). Genetic and epigenetic aspects of type 1 diabetes mellitus: modern view on the problem.. Biomedicines..

[100] Singh R., Chandel S., Dey D. (2020). Epigenetic modification and therapeutic targets of diabetes mellitus.. Biosci Rep..

[101] Arroyo-Jousse V., Garcia-Diaz DF., Codner E., Pérez-Bravo F. (2016). Epigenetics in type 1 diabetes:TNFagene promoter methylation status in Chilean patients with type 1 diabetes mellitus.. Br J Nutr..

[102] Pahkuri S., Ekman I., Vandamme C. (2023). DNA methylation differences within INS, PTPN22 and IL2RA promoters in lymphocyte subsets in children with type 1 diabetes and controls Autoimmunity.. ???.

[103] Cepek P., Zajacova M., Kotrbova-Kozak A., Silhova E., Cerna M. (2016). DNA methylation and mRNA expression of HLA-DQA1 alleles in type 1 diabetes mellitus.. Immunology..

[104] Carry PM., Vanderlinden LA., Johnson RK. (2020). DNA methylation near the INS gene is associated with INS genetic variation (rs689) and type 1 diabetes in the diabetes autoimmunity. study in the young.. Pediatr Diabetes..

[105] Li Y., Zhao M., Hou C. (2011). Abnormal DNA methylation in CD4+ T cells from people with latent autoimmune diabetes in adults.. Diabetes Res Clin Pract..

[106] Miao F., Smith DD., Zhang L., Min A., Feng W., Natarajan R. (2008). Lymphocytes from patients with type 1 diabetes display a distinct profile of chromatin histone H3 lysine 9 dimethylation: an epigenetic study in diabetes.. Diabetes..

[107] Wang Y., Hou C., Wisler J. (2019). Elevated histone H3 acetylation is associated with genes involved in T lymphocyte activation and glutamate decarboxylase antibody production in patients with type 1 diabetes.. J Diabetes Investig..

[108] Kaimala S., Kumar CA., Allouh MZ., Ansari SA., Emerald BS. (2022). Epigenetic modifications in pancreas development, diabetes, and therapeutics.. Med Res Rev..

[109] Hu Q., Che G., Yang Y., Xie H., Tian J. (2020). Histone deacetylase 3 aggravates type 1 diabetes mellitus by inhibiting lymphocyte apoptosis through the microRNA-296-5p/Bcl-xl Axis.. Front Genet..

[110] Dewanjee S., Vallamkondu J., Kalra RS. (2021). The emerging role of HDACs: pathology and therapeutic targets in diabetes mellitus.. Cells..

[111] Kowluru RA., Mohammad G. (2022). Epigenetic modifications in diabetes.. Metabolism..

[112] Margaritis K., Margioula-Siarkou G., Giza S. (2021). Micro-RNA implications in type-1 diabetes mellitus: a review of literature.. Int J Mol Sci..

[113] Ghaffari M., Razi S., Zalpoor H., Nabi-Afjadi M., Mohebichamkhorami F., Zali H. (2023). Association of microRNA-146a with type 1 and 2 diabetes and their related complications.. J Diabetes Res..

